# High-Performance LiNbO_3_ Domain Wall Memory Devices with Enhanced Selectivity via Optimized Metal–Semiconductor Contact

**DOI:** 10.3390/nano14121031

**Published:** 2024-06-14

**Authors:** Haiqing Jiang, Cuihua Dai, Bowen Shen, Jun Jiang

**Affiliations:** School of Microelectronics, Fudan University, Shanghai 200433, China; 22212020009@m.fudan.edu.cn (H.J.); 21112020078@m.fudan.edu.cn (C.D.); 20112020023@fudan.edu.cn (B.S.)

**Keywords:** LiNbO_3_, domain wall, metal-semiconductor contact, ferroelectric memory

## Abstract

Lithium niobate (LiNbO_3_) single-crystal nanodevices featuring elevated readout domain wall currents exhibit significant potential for integrated circuits in memory computing applications. Nevertheless, challenges stem from suboptimal electrode–LiNbO_3_ single crystal contact characteristics, which impact the stability of high currents within these devices. In this work, we concentrate on augmenting the domain wall current by refining the fabrication processes of domain wall random access memory (DWRAM). Each LiNbO_3_ domain wall nanodevice was fabricated using a self-aligned process. Device performance was significantly enhanced by introducing a 10 nm interlayer between the LiNbO_3_ and Cu electrodes. A comparative analysis of electrical properties was conducted on devices with interlayers made of chromium (Cr) and titanium (Ti), as well as devices without interlayers. After the introduction of the Ti interlayer, the device’s coercive voltage demonstrated an 82% reduction, while the current density showed a remarkable 94-fold increase. A 100 nm sized device with the Ti interlayer underwent positive down–negative up pulse testing, demonstrating a writing time of 82 ns at 8 V and an erasing time of 12 μs at −9 V. These operating speeds are significantly faster than those of devices without interlayers. Moreover, the enhanced devices exhibited symmetrical domain switching hysteresis loops with retention times exceeding 10^6^ s. Notably, the coercive voltage (*V*_c_) dispersion remained narrow after more than 1000 switching cycles. At an elevated temperature of 400 K, the device’s on/off ratio was maintained at 10^5^. The device’s embedded selector demonstrated an ultrahigh selectivity (>10^6^) across various reading voltages. These results underscore the viability of high-density nanoscale integration of ferroelectric domain wall memory.

## 1. Introduction

In the field of integrated circuits for in-memory computing applications, there is a pressing demand for high-speed, low-power, non-volatile memory for the exponential growth of artificial intelligence [1,2,3,4,5]. Extensive research has been devoted to emerging non-volatile memory technologies, such as resistance-based random access memory (RRAM), magnetic random access memory (MRAM), and phase-change random access memory (PRAM). Despite their unique advantages, the inherent stochasticity of switching processes in PRAM and RRAM leads to limited durability, low operating speed, and poor retention [6,7,8], whereas MRAM exhibits high write energy consumption [9]. Employing a single transistor as a memory cell, non-volatile ferroelectric field effect transistors (FeFETs) offer an advantage over conventional FeRAM in both the 1T−1C (1 transistor−1 capacitance) and 2T−2C (2 transistor−2 capacitance) configurations. This advantage stems from their ability to maintain polarization direction during read operations, enabling non-destructive readout of stored data via the detection of on/off channel currents [10,11,12]. Despite the simplicity of its structure and the improved storage density resulting from the reduced 1T (1 transistor) footprint per memory cell, short retention time, leakage current, and operational lifetime remain significant challenges that hinder the practical implementation of this technology [13].

In contrast, recent years have witnessed a surge of interest in the development of ferroelectric-based domain wall random access memory (DWRAM), which offers a compelling combination of non-volatility, high-speed operation, low power consumption, enhanced reliability, and excellent scalability. These advancements position DWRAM as a promising candidate for next-generation general-purpose memory applications [14,15,16,17,18,19]. Seidel et al. first reported pA-scale currents flowing along 109° and 180° domain walls in BiFeO_3_ thin films using an atomic force microscope tip [20]. Following this discovery, numerous reports have been published in the literature documenting similar observations of domain wall currents in various ferroelectric materials [18,21,22,23,24,25,26]. The magnitude of the domain wall current is insufficient to meet the demands of high-speed reading circuits [27,28]. The potential for ferroelectric domain wall random access memory (DWRAM) applications emerged with the performance improvement that domain wall currents in LiNbO_3_ (LNO) could reach the order of microamperes [29,30]. In single-crystal LNO thin films, bipolar domain orientations can be exhibited to represent digital “1” and “0” data by creating and erasing domain walls (DWs) between two antiparallel and parallel domains, respectively. Notably, the domain wall current in this system can reach the order of several microamperes [31,32,33,34,35]. 

Despite these promising advancements, several challenges remain in the path towards the commercialization of DWRAM technology [25]. Among these challenges, the most critical is the need to increase the domain wall (DW) current density in nanodevices to drive fast memory circuits at low read voltages [20,29,36,37]. The presence of interfacial layers between the memory cell and electrodes, which impede the passage of a substantial domain wall (DW) current through the LNO cell, is hypothesized to be the underlying cause of the low DW current density observed in nanodevices [38]. In addition, ferroelectric memory devices are susceptible to a detrimental effect known as an imprint effect. This phenomenon is characterized by the gradual displacement of the hysteresis loop along the voltage axis over time. Imprint can result in an asymmetry in the coercive field, destabilization of one of the polarization states, and ultimately lead to write failure or data retention loss [39,40,41]. The electrical properties of ferroelectric memory devices are significantly influenced by the selection of electrode materials that come into contact with the ferroelectric layer. In particular, ferroelectric memory devices based on hafnium oxide (HfO_2_) have been extensively studied to optimize electrode materials. Experimental results have demonstrated that the electrode material can modify the oxygen vacancy (O-phase) ratio and system energy of the ferroelectric material, thereby affecting the retention properties of HfO_2_-based ferroelectric memories [1]. However, limited research has been conducted on the optimization of electrode materials for LNO memory devices.

In this work, we significantly enhanced the domain wall (DW) current in LNO DWRAM devices by refining the fabrication processes. Each LNO DW nanodevice was fabricated in self-aligned contact with two side electrodes, following complementary metal–oxide–semiconductor (CMOS) fabrication processes. To optimize the metal–semiconductor contact, a 10 nm interlayer was introduced. During the writing and erasure process, the application of in-plane positive/negative external electric fields to the two side electrodes induces the formation of antiparallel/parallel domains between the LNO cell and the underlying substrate. This enables the formation or erasure of conducting domain walls between them. The coercive voltage and current of devices with and without interlayers were analyzed and compared. The introduction of interlayers effectively mitigates the imprint effect. All devices exhibit stable electrical performance and high retention.

## 2. Experimental Methods

**Figure 1:**
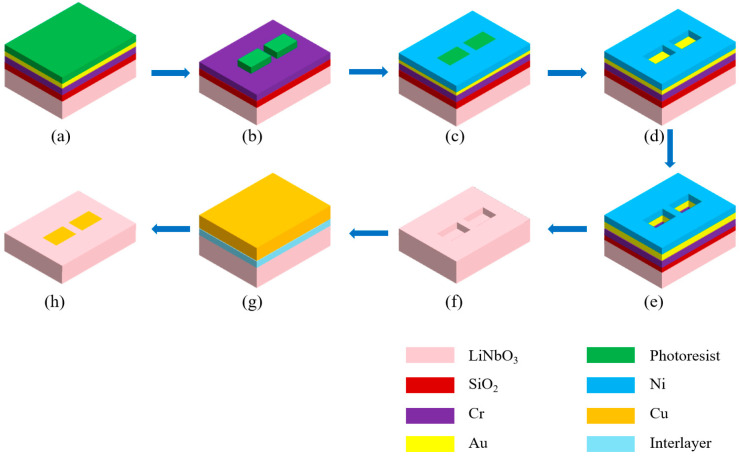
Fabrication processes for the LiNbO_3_ memory devices. (**a**) Deposited SiO_2_, Cr, and Au as a seek layer and spin-coated photoresist on the surface of the Au layer. (**b**) Exposed mark area after EBL patterning. (**c**) Electroplated Ni layer on the seed layer without the photoresist. (**d**) Removed photoresist. (**e**) Etched Au, Cr, SiO_2,_ and LiNbO_3_ using RIE. (**f**) Removed Ni, Au, Cr and SiO_2_. (**g**) Deposited interlayer and Cu. (**h**) Removed Cu outside the trench area using CMP.

Nanodevice Fabrication. X-cut LNO single crystals containing 48.5 mol % Li_2_O with a 5 mol % MgO dopant were grown using the Czochralski technique with high-purity (99.99%) Li_2_CO_3_, MgO, and Nb_2_O_5_ powders that were melted at 1250 °C. A regulated current density of around 50 mA/cm^2^ was applied to polarize the crystal for 30 min at a Curie temperature of 1160–1210 °C to create a ferroelectric single-domain pattern. After cutting off the crystal into 10 × 12 × 0.5 mm^3^ pieces, a 100 nm thick SiO_2_ layer was deposited at 400 °C on the surface of LNO substrates using plasma-enhanced chemical vapor deposition (PECVD) to enhance the adhesion of subsequent metal layers to the LNO substrate, 10 nm thick Cr films and 30 nm thick Au were then grown using magnetron sputtering (PVD-75, Kurt J. Lesker, Jefferson Hills, PA, USA) at room temperature as a seed layer (for subsequent electroplated Ni, where Cr improved the adhesion between Au and SiO_2_). A 130 nm thick poly (methyl methacrylate) photoresist layer was spin-coated on the LNO surface, and the sizes of LNO mesa-cells were then defined and patterned using electron beam lithography (EBL JEOL 6300FS, Tokyo, Japan) (Figure 1a,b). The 100 nm thick Ni layer was electroplated on the exposed LNO area seed layer without the photoresist as a hard mask, the remaining photoresist was removed by acetone (Figure 1c,d). The area that was not protected by the Ni layer was etched to a depth of 260 nm using reactive ion etching (RIE) (SAMCO Corporation, Kyoto, Japan) (Figure 1e). After cleaning off the etching residuals, Ni, Cr, Au and SiO_2_ layers were removed (Figure 1f). Finally, a 10 nm think interlayer and a 400 nm thick Cu layer were deposited by magnetron sputtering and formed two side electrodes to contact each LNO mesa after chemical–mechanical polishing (CMP) (Figure 1g,h).

Domain Imaging and Electrical Characterization. Images from a field emission scanning electron microscope (SEM, Sigma HD, Zeiss, Jena, Germany) were used to analyze all mesa-like memory cells with dimensions of *w* × *l* × *h* (width × length × height). Using in-plane piezoelectric force microscopy (PFM) amplitude and phase imaging (Icon, Bruker, Billerica, MA, USA) with a contact Pt Ir-coated silicon tip with a radius of about 20 nm and a force constant of 2.8 N/m with an AC amplitude of 2.5 V at 230 kHz, the written domain patterns using positive/negative poling voltages were examined. When performing PFM imaging of the inverted domains, the high characteristic frequency can lessen the artifact of the injected charge during the long relaxation time. A voltage-sweep-mode-equipped Agilent B1500A (Santa Clara, CA, USA) semiconductor analyzer (Keysight, San Francisco, CA, USA) was used to measure each double current–voltage (*I*–*V*) curve. With an instrumental off current resolution of 20 pA and a current amplification range of 100 μA, the sweep times were 1 s. Using a two-channel Agilent 81110A pulse generator, square pulses with rising times of 2 ns were applied for domain switching testing. An oscilloscope (HDO6054, LeCroy, Chestnut Ridge, NY, USA) with a 12-bit voltage resolution and a 1 GHz bandwidth was used to directly examine the steady-state domain wall current transient with time. The oscilloscope’s internal resistance in series with the sample was first set to 50 Ω to determine the short-circuit RC time constant during the domain switching period. Later, it was adjusted to 1 MΩ to allow for the readout of on/off currents with a resolution limit of 1 nA.

Device simulation. TCAD simulations were performed on Silvaco.2020.win64 (Santa Clara, CA, USA) using Athena and Atlas tools. The device simulation parameters are selected such that they match with the experimental data, viz., thickness, lengths, widths, and work functions, etc. The standard Poisson charge transport equations were solved using the Newton method.

## 3. Results and Discussion

**Figure 2:**
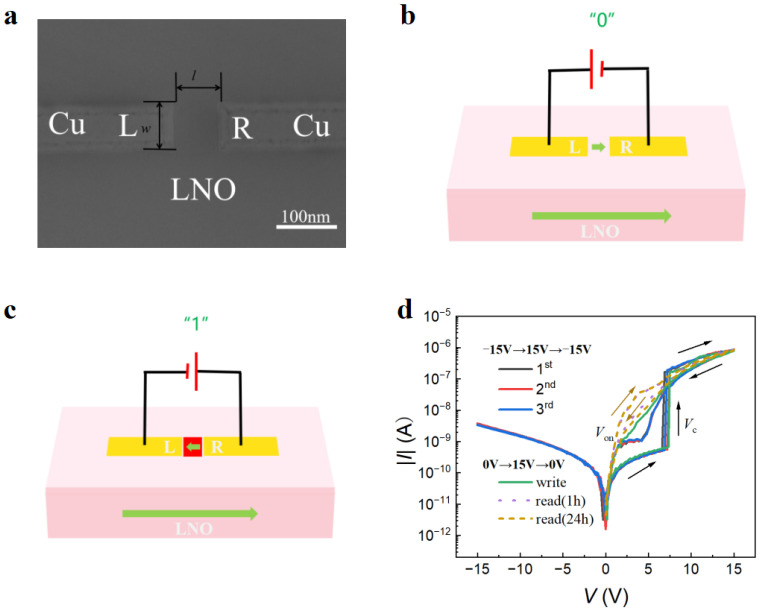
(**a**) Planar SEM image of a typical two-terminal domain wall nanodevice (*w* × *l* × *h* = 100 × 80 × 90 nm^3^) with Cu electrode. (**b**,**c**) Schematic diagrams of experimental setups and the working principle of an LiNbO_3_ memory cell during the writing of “0” and “1” data, respectively. (**d**) Double *I*–*V* curves in different voltage sweeping ranges of a domain wall nanodevice (*w* × *l* × *h* = 100 × 80 × 90 nm^3^) with Cu electrode in a semi-logarithmic plot. Thin arrows show voltage sweeping directions.

Figure 2a shows a typical planar scanning electron microscope (SEM) image of a two-terminal domain wall memory device (*w* × *l* × *h* = 150 × 100 × 90 nm^3^) in contact with two Cu electrodes. The electrode width (*w*) and the gap distance (*l*) between the left and right side electrodes (L and R), respectively, are indicated in the image. Figure 2b,c illustrate the electrical setups for the LNO DWRAM. The Cu electrodes are represented by the yellow regions, and the LNO is represented by the pink regions. Thick arrows indicate the directions of polarization. When the applied voltage causes the polarization direction of the LNO between the electrodes to differ from that of the substrate, a conductive DW can be generated. The conductivity of the DW region is approximately 10^3^–10^6^ times higher than that of the insulating region [42,43,44]. Figure 2d shows the double *I*–*V* curves after various sweeping cycles for memory cells (*w* × *l* × *h* = 100 × 80 × 90 nm^3^) with Cu electrodes. During the first *I*–*V* sweep from −15 to +15 V (arrows indicate the voltage sweeping directions), the initial off currents abruptly switched to an on current of 0.38 μA above a coercive voltage (*V*_c+_) of 6.9 V. This indicates the formation of conductive domain walls upon domain switching. The red area in Figure 2c represents the reversed domain and the conductive domain walls between them. Conversely, the reversed domains revert into their starting states with the erasure of the conductive domain walls (parallel domains in Figure 2b) when the applied voltage is swept back below a negative coercive voltage (*V*_c−_), and as a result, the wall currents between L and R switch to an off state. In the next repeated cycles (second and third sweeps), *V*_c+_ reduces a little and gradually levels off. During the fourth *I*–*V* sweep from 0 to 15 V in Figure 2d, the information “1” was written by inducing the formation of a conductive domain wall. The information “1” may be read out at a read voltage ranging from 0 to *V*_c+_, as verified by the fifth and sixth *I*–*V* sweep from 0 to 15 V, which occurred after 1 h and 24 h, respectively. During the fifth and sixth voltage sweeps shown in Figure 2d, the DW current is always on when the applied voltage is greater than an onset voltage (*V*_on_). Previous studies have shown that a “dead” layer exists at the interface between the electrode and LNO, and that when the write voltage is withdrawn, the domains within the dead layer become volatile [35,38]. In Figure 2d, a diode-like DW current is rectified by the volatile interfacial domains, which disconnect the inner non-volatile domain wall (encoding “1” and “0” information) from the L and R electrodes at negative applied voltages. When the applied voltage exceeds *V*_on_, the interfacial domain wall reconnects L and R, allowing a read current to flow. Volatile interfacial domains can effectively mitigate crosstalk and leakage currents between devices within a crossbar array [43].

**Figure 3:**
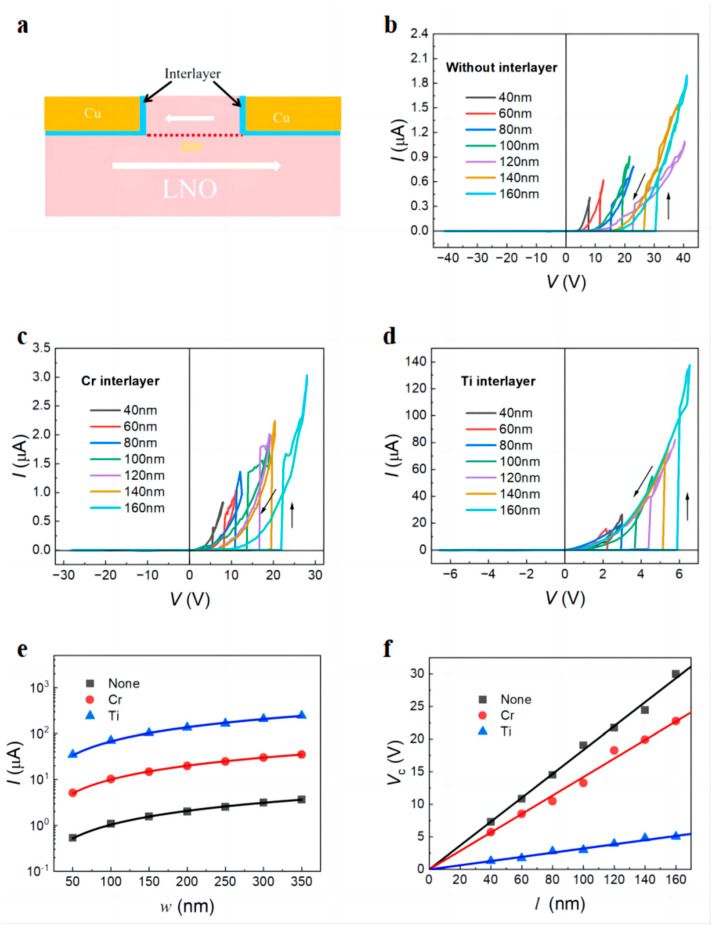
(**a**) Schematic diagram of introducing a 10 nm interlayer at the metal–semiconductor contact of the domain wall memory nanodevice. (**b**–**d**) Double *I*–*V* curves during the first voltage sweeps for LiNbO_3_ nanodevices at different *l* when *w* = 150 nm and *h* = 90 nm without interlayers and with different interlayers. (**e**) Electrode width dependence of on current at 8 V without and with different interlayers when *l* = 100 nm and *h* = 90 nm fitted by the solid lines. (**f**) Gap length dependence of coercive voltage without and with different interlayers when *w* = 150 nm and *h* = 90 nm fitted by the solid lines. The parenthetic values show the slopes of the linear fits. Thin arrows show voltage sweeping directions.

Figure 3a shows a schematic diagram of the device with a 10 nm interlayer. Due to the high density of surface states on the LNO surface and the high-resistance interface layer of CuO formed by oxygen atoms in LNO and the Cu electrode, direct contact between LNO and Cu will generate a large contact barrier, resulting in a larger Schottky contact resistance [45]. To optimize the metal–semiconductor contact, a 10 nm interlayer was deposited (300 °C, 1 min) and then treated in an ultra-high vacuum environment at 300 °C for 0.8 h. Figure 3b–d show the *I*–*V* curves of devices without and with different interlayers at different *l* values when *w* = 150 nm and *h* = 90 nm. When *V* < *V*_c_, the device is in the off state and the current is on the nA scale. When *V* > *V*_c_, the off current jumps to the on current. The on current is the diode type. The on current of devices without interlayers is 0.3–2 μA, and the coercive voltage is distributed between 8.1 and 30.3 V for devices with different *l* values. The on current of devices with Cr interlayers is 1.1–3 μA, and the coercive voltage is distributed between 5.6 V and 22.1 V for devices with different *l* values. The on current of devices with Ti interlayers is 23.1–133.2 μA, and the coercive voltage is distributed between 2.2 and 5.9 V for devices with different *l* values. Figure 3e shows that the on current is proportional to the electrode width *w* when *l* = 100 nm and *h* = 90 nm, which can be explained by the space charge limitation model [38]. From the slopes of the three fits, we calculated the linear current density of 45.9 μA/μm for the memory devices with Ti interlayers which is 94 times that of devices without interlayers and 65 times that of devices with Cr interlayers. As shown in Figure 3f, the *V*_c_ almost linearly changes with the increase in *l* when *w* = 100 nm and *h* = 90 nm. From the slopes of two solid lines, we estimated their positive coercive fields as 561, 1827, and 1433 kV/cm, respectively. Therefore, the coercive field can be lowered by 22% for the memory devices with Cr interlayers and by 82% for the memory devices with Ti interlayers, which enables the low-voltage operation of the DWRAM. 

**Figure 4:**
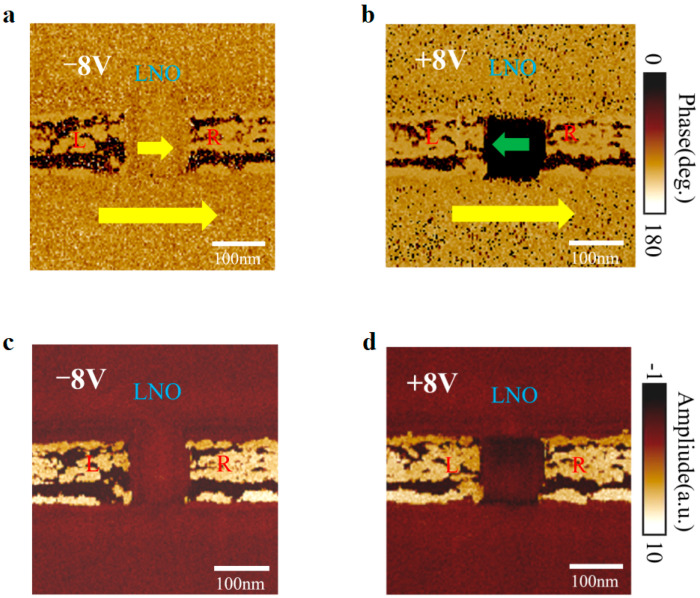
Domain wall formation. (**a**,**c**) In-plane PFM phase and amplitude images of switched domains (*w* × *l* × *h* = 100 × 80 × 70 nm^3^) after poling voltage of −8 V for the LiNbO_3_ device with a Ti interlayer. The yellow/green arrows show polarization orientations before/after domain switching, L and R represent the left and right electrode regions on both sides. (**b**,**d**) In-plane PFM phase and amplitude images of switched domains (*w* × *l* × *h* = 100 × 80 × 70 nm^3^) after poling voltage of 8 V for the device with a Ti interlayer.

To unravel the domain switching behavior, we observed the PFM images of the device at different poling voltages. Figure 4a,c show the in-plane PFM phase and amplitude images of the device (*w* × *l* × *h* = 100 × 80 × 70 nm^3^) with a Ti interlayer under an applied poling voltage of −8 V. The LNO region between L and R has a polarization direction parallel to the underlying substrate’s polarization, and the device is in the off state with no phase transition in the region between L and R. When the polarization voltage is higher than the positive coercive voltage (*V*_c+_), for the device (*w* × *l* × *h* = 100 × 80 × 70 nm^3^), antiparallel domains appear in the region between L and R. The device transitions from an off state to an on state due to the appearance of domain walls with high conductivity, as shown in Figure 4b. The PFM image shows a 180-degree phase change under the application of a write voltage of 8 V. Meanwhile, we observed the DW region (two black thin lines between L and R) through the PFM amplitude images in Figure 4d.

**Figure 5:**
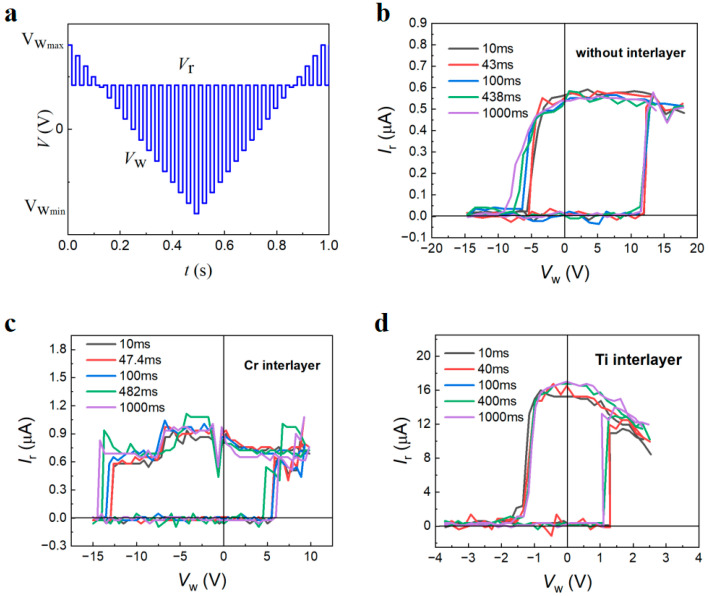
(**a**) Interweaved write–read voltage pulses for the characterization of *I*_r_−*V*_w._ hysteresis loops. (**b**–**d**) *I*_r_−*V*_w_ hysteresis loops at various periodicities for the LiNbO_3_ nanodevice (*w* × *l* × *h* = 150 × 100 × 90 nm^3^) without interlayers and with different interlayer at read voltages of 0.5 V.

As shown in Figure 5a, a triangular write pulse was applied to the devices without and with different interlayers to study the dynamics of domain flipping. To read out the on/off currents (*I*_r_), a read voltage of 0.5 V (*V*_on_ < *V*_r_ < *V*_c+_) was applied between two adjacent writing voltages (*V*_w_). *I*_r_−*V*_w_ hysteresis loops at varied periodicities for the devices (*w* × *l* × *h* = 150 × 100 × 90 nm^3^) without and with different interlayers are displayed in Figure 5b–d. We calculated *V*_c+_ and *V*_c−_ from the off-to-on and on-to-off current jumps, respectively. With the decreased periodicity, both |*V*_c+_| and |*V*_c+_| increased, indicating frequency-dependent domain switching behavior. For the device without interlayers, |*V*_c+_| is higher than |*V*_c−_|. Conversely, for the devices with Cr interlayers, |*V*_c−_| is higher than |*V*_c+_|. The imprint effect causes the loops in both the Cr interlayer and the non-interlayer devices to be asymmetric [41,46]. However, for the devices with Ti interlayers, at periodicities below 100 ms, the loops become symmetric due to the diffusion of Ti atoms into LiNbO_3_, which can increase the conductivity of the interface, thereby effectively withdrawing the injected space charge and screening the depolarization field near the interface to mitigate the adverse effects of imprint effect.

**Figure 6:**
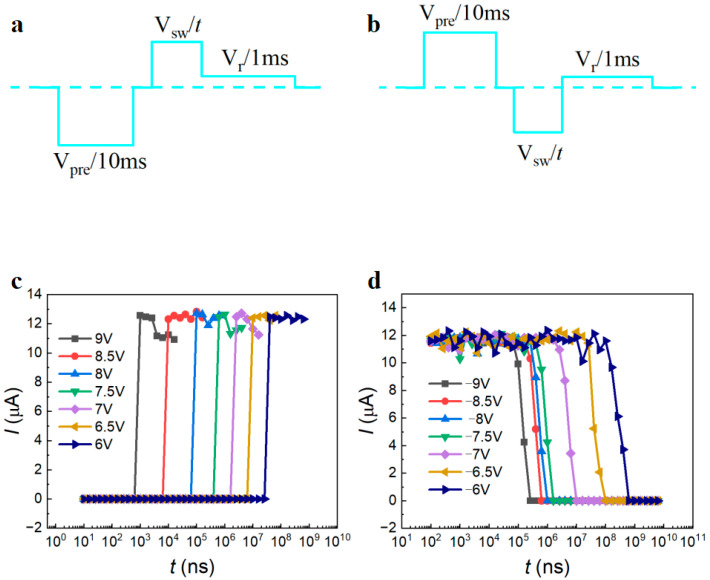
(**a**,**b**) Schematic of pulse sequences for testing of forward and backward domain switching times under applied positive and negative switching voltages, respectively. (**c**,**d**) Write time dependences of *I*r at 3.5 V after different positive and negative write voltages for the LiNbO_3_ nanodevice (*w* × *l* × *h* = 100 × 120 × 70 nm^3^) with a Ti interlayer.

According to the Johnson–Nyquist limit, a readout current greater than 1 μA is required to read the logic information stored in the circuit within 10 ns [47]. The positive and negative domain switching periods of the device (*w* × *l* × *h* = 100 × 120 × 70 nm^3^) with a Ti interlayer are measured using the two pulse sequences in Figure 6a,b, which have opposite polarity. Domain switching voltage (*V*_sw_) at pulse width *t* is applied after the application of a negative/positive presetting pulse (*V*_pre_) with a width of 10 ms. Once the on/off currents are determined, a read pulse with a width of 1 ms and a voltage of *V*_r_ = 0.5 V is applied. The temporal variations of the read current at different write voltages are shown in Figure 6c,d. The domain switching time, which is voltage-dependent and characterized by the Merz law, is determined from the off-to-on/on-to-off current jumps [48]. The writing time is approximately 80 ns at 8 V, and the erasing time is 23 μs at −8 V. However, under the same applied electric field, the switching speed in the negative domain is nearly three orders of magnitude slower than in the positive domain. This phenomenon can be explained by space charge injection, as thorough charge injection along the DWs can produce an internal screening electric field [49].

**Figure 7:**
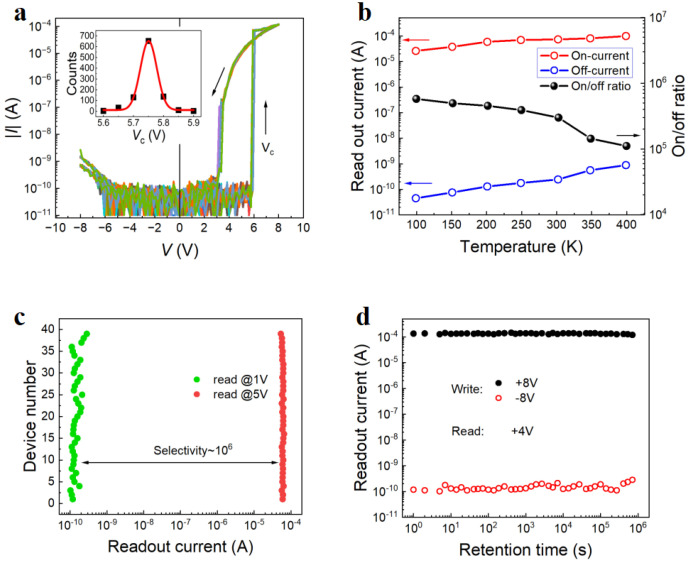
(**a**) One thousand sweeps of *I*–*V* curves for the LiNbO_3_ nanodevice (*w* × *l* × *h* = 100 × 80 × 90 nm^3^) with a Ti interlayer in a semi-logarithmic plot, where the thin arrows show voltage sweeping directions and the inset show statistical *V*_c+_ distributions fitted by the solid lines. (**b**) The temperature dependence of the on currents, off currents, and on/off ratios at 3.5 V for the LiNbO_3_ nanodevice (*w* × *l* × *h* = 100 × 80 × 90 nm^3^) with a Ti interlayer in a semi-logarithmic plot. (**c**) Statistical distribution of readout currents for various fresh LiNbO_3_ nanodevices (*w* × *l* × *h* = 100 × 80 × 90 nm^3^) with a Ti interlayer measured at 1 V and 5 V, respectively. (**d**) Retention time depends on on currents and off currents at 3 V after different write voltages for the LiNbO_3_ nanodevice (*w* × *l* × *h* = 100 × 140 × 90 nm^3^) with a Ti interlayer.

The repetitive domain expansion under the applied *V* is highly repeatable and has good retention capability. Figure 7a shows 1000 sweeps of *I*–*V* curves between −8 and 8 V in a semi-log plot for a nanodevice (*w* × *l* × *h* = 100 × 80 × 90 nm^3^) with a Ti interlayer. The insets display the statistically coercive voltage distributions fitted by the Gaussian function. The *V*_c+_ dispersion for the nanodevice is only within 0.3 V. Figure 7b shows the temperature dependence of on currents, off currents, and on/off ratios in a semi-log plot for a nanodevice (*w* × *l* × *h* = 100 × 80 × 90 nm^3^) with a Ti interlayer. Both the device’s on currents and off currents increased with increasing temperature, with the off currents exhibiting a more pronounced increase. This behavior can be attributed to thermally excited carriers having a greater impact on the off current than on the on current. Notably, despite the decrease in the on/off ratio of the device with increasing temperature, the on/off ratio remained as high as 10^5^, even at 400 K. The LNO DW devices have an embedded interfacial layer selector [41]. To study the selectivity of the device, we applied a write voltage of 8 V to different devices with Ti interlayers and read the device’s current at 1 V and 5 V, respectively. The statistical distribution of the current in Figure 7c indicates that for various devices (*w* × *l* × *h* = 100 × 80 × 90 nm^3^) with Ti interlayers, the readout currents at 1 V (<*V*_on_) mostly varied from 0.06 to 0.2 nA, whereas the readout currents at 5 V (>*V*_on_) almost stayed constant. There is a 10^6^ times difference between the two currents, reflecting the extremely excellent selectivity of the LNO DW device, which can meet almost all memory circuits that require selectors. This phenomenon may be attributed to the fact that single crystals of LiNbO_3_-containing Ti atoms exhibit enhanced control over the reconnection of domain walls at the interface with the L and R electrodes. Retention time dependences of the on and off currents at 4 V of devices (*w* × *l* × *h* = 100 × 80 × 90 nm^3^) with Ti interlayers after various write voltages are shown in Figure 7d. When considering retention time, both currents exhibit stability when the on/off ratio exceeds 10^6^ s. 

**Figure 8:**
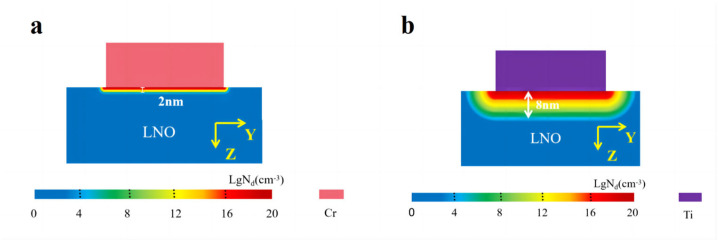
(**a**) The spatial Cr atoms distribution after depositing a 10 nm Cr layer (300 °C, 1 min) on a LiNbO_3_ cell (*w* × *l* × *h* = 50 × 40 × 20 nm^3^) and placing it in an ultra-high vacuum environment at 300 °C for 60 min. (**b**) The spatial Ti atoms distribution after depositing a 10 nm Ti layer (300 °C, 1 min) on a LiNbO_3_ cell (*w* × *l* × *h* = 50 × 40 × 20 nm^3^) and placing it in an ultra-high vacuum environment at 300 °C for 60 min.

For the understanding of the Cr and Ti interlayer effect on the polarization retention and coercive voltage, the spatial metal atoms distribution was simulated for the LNO cells (*w* × *l* × *h* = 50 × 40 × 20 nm^3^). Our primary concern was the diffusion of metal atoms parallel to the domain wall direction (i.e., the Z direction). Figure 8a,b show the depth dependence of the simulated metal atoms distribution under the deposited 10 nm layer (300 °C, 1 min) after the treatment in an ultra-high vacuum environment at 300 °C for 60 min. For the cell deposited with a Cr layer, the layers with a thickness of 2 nm are rich (>10^8^ cm^−3^) with Cr atoms. For the cell deposited with a Ti layer, the layers with a thickness of 8 nm are rich (>10^8^ cm^−3^) with Ti atoms. The layers rich with metal atoms can work as heavily doped n-type semiconductors, forming nearly Ohmic contacts between the Cu electrodes and the LNO film, assisting in reducing the coercive field and increasing the DW current.

## 4. Conclusions

We demonstrated a method to improve the readout current density and selectivity of LNO devices by optimizing metal–semiconductor contact. By improving the devices’ manufacturing processes, we were able to significantly raise the wall current. Every LNO DW nanodevice in contact with two side electrodes was constructed via self-alignment in compliance with CMOS fabrication techniques. We deposited a 10 nm interlayer between LNO and Cu electrodes. The electrical properties of devices without interlayers, as well as those with interlayers of Cr and Ti, were compared. After the introduction of a Ti interlayer, the device’s coercive field decreased by 82%, while the current density increased by 94 times. Subsequent positive down–negative up pulse tests of a 100 nm sized device with a Ti interlayer revealed a writing time of 82 ns at 8 V and an erasing time of 12 μs at −9 V, which is significantly quicker than the operation rates of a device without interlayers. Furthermore, symmetrical domain switching hysteresis loops with retention times longer than 10^6^ s were observed in the improved devices. The *V*_c_ dispersion is very narrow after a switching number of more than 1000 cycles. The on/off ratio of the device can maintain 10^5^ at 400 K. Polarized devices exhibit selectivities of 10^6^ at different reading voltages. The fabrication process of the devices is CMOS-compatible, paving the way for future high-density DWRAM.

## Data Availability

Data are contained within the article.
